# Long-term follow-up and novel splice donor mutation in *MEN1* in a Chinese family

**DOI:** 10.18632/oncotarget.23100

**Published:** 2017-12-07

**Authors:** Minghao Li, Qianqian Liu, Peihua Liu, Xiaoping Yi, Xiao Guan, Anze Yu, Longfei Liu, Feizhou Zhu

**Affiliations:** ^1^ Department of Urology, Xiangya Hospital, Central South University, Changsha, China; ^2^ Department of Biochemistry and Molecular Biology, School of Life Sciences, Central South University, Changsha, China; ^3^ Department of Radiology, Xiangya Hospital, Central South University, Changsha, China; ^4^ Xiangya Hospital, Central South University, Changsha, China

**Keywords:** multiple endocrine neoplasia type 1, MEN1 gene, splicing site mutation, somatic mutation

## Abstract

Heterozygous germline mutation of the *MEN1* tumor suppressor gene is responsible for multiple endocrine neoplasia type 1. Parathyroid and thoracic neuroendocrine tumor specimens and DNA from two Han Chinese MEN1 family patients were analyzed using whole exome and Sanger sequencing. The proband (II-3) was sequentially diagnosed with pituitary adenoma, pancreatic tumor, adrenal cortical tumor, abdominal lipoma, and parathyroid adenoma during the 6-year follow-up. The son of the proband (III-6) was also diagnosed with a thoracic neuroendocrine tumor and a parathyroid adenoma during this period. Splice alterations were studied by RT-PCR and sequencing. The mutation impact was evaluated using bioinformatics. Sequence analysis revealed a novel splice donor mutation, *MEN1* IVS9 + 1G > C, that changed the splicing mode of MEN1 to halt translation before two nuclear localization signals in the menin protein. Novel somatic mutations, *MEN1* c.1402_1405delGAGG and c.286 C > T, were identified in the parathyroid adenoma of II-3 and thoracic neuroendocrine tumor of III-6, respectively, indicating a two-hit etiology of MEN1 syndrome. Our study revealed the clinical and genetic basis of MEN1 in this Han Chinese family and provides insight into MEN1 mechanisms, diagnosis, and management.

## INTRODUCTION

Multiple endocrine neoplasia type 1 (MEN1) syndrome is an autosomal dominant disease characterized by the occurrence of tumors involving two or more endocrine glands within a single patient [1]. The estimated worldwide prevalence of MEN1 is between 1/30000 and 1/500000 [1, 2]. More than 1000 families presenting with MEN1 syndrome have been described since the cloning of the *MEN1* gene in 1997 [3]. However, clinical treatment and long-term follow-up are rarely reported in the literature. MEN1-associated tumors usually occur in multiples, readily metastasize, involve multiple organs, and are larger than sporadic endocrine tumors. The treatments for each type of MEN1-associated endocrine tumor are similar to those of non-MEN1 patients, but the outcomes of MEN1-associated tumors are less successful [1]. Therefore, a thorough understanding of clinical features with long-term follow-up data is essential for the management of MEN1.

Inactivation of the *MEN1* tumor suppressor gene causes MEN1 [4]. *MEN1* is localized to chromosome 11q13 and encodes the 610 or 615 amino acid nuclear protein, menin. Menin is a widely expressed protein that consists of three nuclear localization signals (NLSs) and five putative GTPase sites. Menin interacts with proteins involved in transcriptional regulation, genome stability, cell division, and proliferation, but its function is still poorly understood [5]. More than 600 different germline mutations and 160 different somatic mutations have been identified in MEN1 patients [3, 6]. The splice-site mutation rate was 9% among these mutations. The significance of splicing donor mutations is insufficiently reported in the literature.

A novel germline-splicing donor mutation, *MEN1* IVS9 + 1G > C, was identified in a Han Chinese MEN1 family with a 6-year follow-up period and its functions were investigated. Two novel somatic mutations, *MEN1* c.1402_1405delGAGG and c.286 C > T, and loss of IVS9 + 1G > C heterozygosity (LOH) were found in MEN1 tumor tissues. Here, we elucidated the relationship between clinical features and *MEN1* mutations in a Han Chinese MEN1 family and enriched the theoretical basis of MEN1 clinical diagnosis and therapy.

## RESULTS

### Extended family presentation of MEN1 syndrome

**Figure 1:**
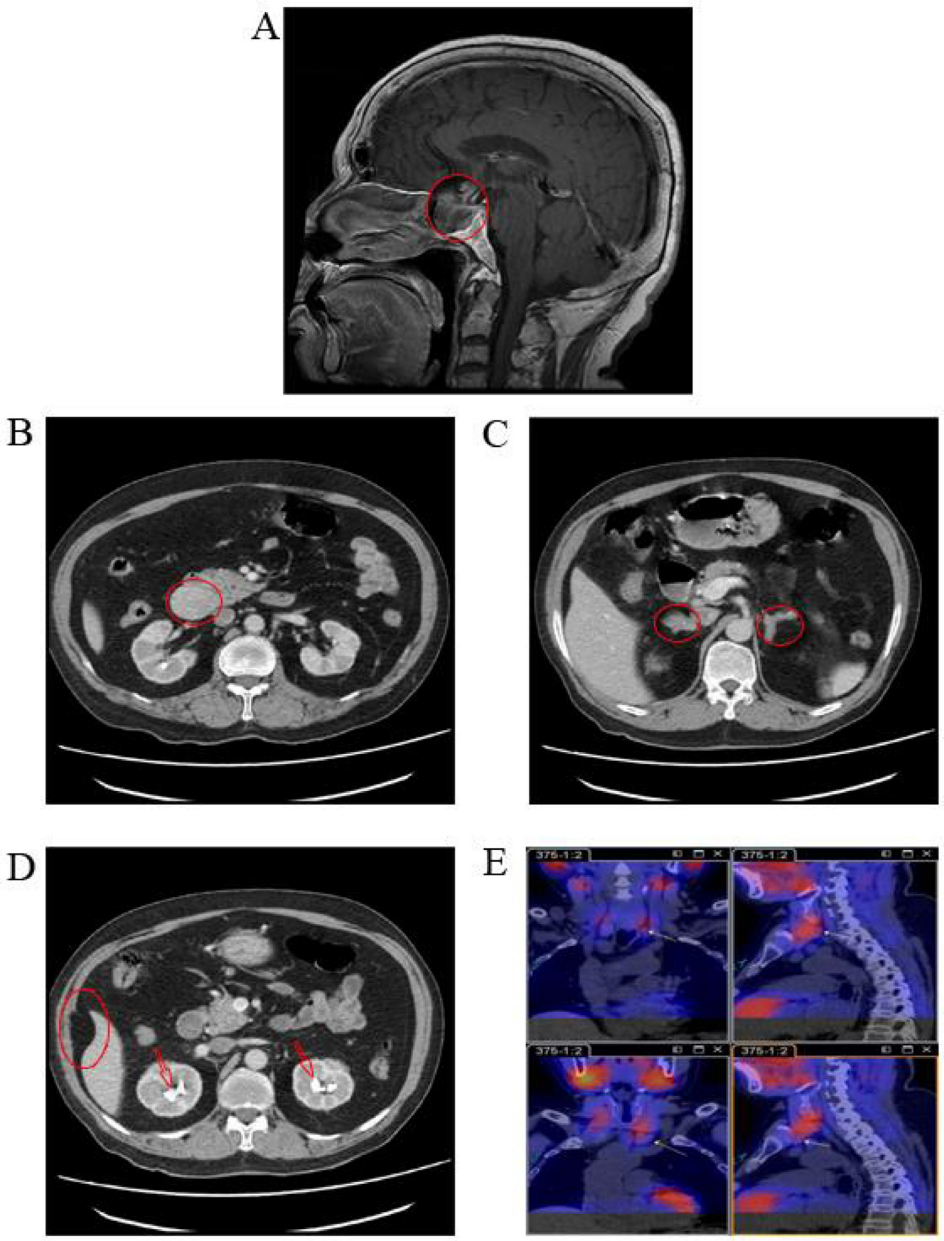
Medical imaging of the proband, II-3, in the *MEN1* syndrome family (**A**) MRI scans revealed a 2.0 × 2.5 × 2.5 cm pituitary tumor in the proband; (**B**, **C**, and **D**) Abdominal CT revealed a solid mass in the pancreatic head, bilateral adrenal nodules, bilateral renal stones, and abdominal lipoma in the proband; (**E**) SPECT revealed multiple nodules behind thyroid in the proband. MRI, magnetic resonance imaging; CT, computed tomography; SPECT, single photon emission computed tomography.

The proband (II-3), a male born in December 1945, visited Xiangya Hospital in August 2010 for dizziness. Magnetic resonance imaging (MRI) revealed a 2.0 × 2.5 × 2.5 cm pituitary tumor (Figure 1A) that was presumed to be a functional pituitary adenoma due to the high serum prolactin level (Table 1). The patient refused surgical treatment. Therefore, 10 mg/day of bromocriptine was administered, and regular follow-up visits were instituted. Subsequent examinations indicated that the pituitary adenoma had been controlled. In March 2014, the patient revisited Xiangya Hospital for epigastric pain, sicchasia, and vomiting. Abdominal computed tomography (CT) revealed a 4 × 3 cm solid mass localized in the pancreatic head, bilateral renal stones, bilateral adrenal nodules, and abdominal lipoma (Figure 1). Cervical ultrasonography revealed a left 8 × 5 mm and a right 9 × 5 mm auxetic parathyroid nodule. Barium meal examination revealed chronic non-atrophic gastritis and multiple polyps of the duodenal bulb (data not shown). The pancreatic tumor was surgically resected and histologically diagnosed as a pancreatic neuroendocrine tumor (data not shown). The blood biochemistry prolactin and parathyroid levels were outside the normal range (Table 1). A patient with 2 or more MEN1-associated tumors should be diagnosed with MEN1 according to the clinical diagnostic criteria [7]. In August 2016, the patient returned to Xiangya Hospital as the parathyroid nodule was enlarging progressively. Single-Photon Emission Computed Tomography (SPECT) revealed multiple nodules behind the thyroid (Figure 1). The parathyroid tumors were resected and diagnosis of adenoma with lymphatic metastasis was confirmed by pathology (data not shown).

**Table 1:** Blood biochemistry profiles of the proband from 2010

Biochemical molecule	Oct. 2010	Mar. 2014	Aug. 2016	Reference range
PRL (ng/mL)	1194.00	306.50	1035.11	4.04–15.2
PTH (pg/mL)	NA	1437.00	1458.00	15–65
TSH (mIU/L)	1.150	3.300	2.440	0.27–4.2
FSH (IU/L)	2.23	8.57	7.54	1.5–12.4
LH (IU/L)	1.80	8.83	7.86	1.7–8.5
TESTO (ng/mL)	0.414	1.510	1.840	2.8–8.0
ACTH (pmol/L)	7.82	0.29	NA	1.6–13.9
Cortisol, 4 PM (μg/dL)	14.05	10.40	NA	2.3–11.9
E2 (pmol/L)	NA	96.900	101.700	28–156
PROG (ng/mL)	NA	0.17	0.16	0.2–1.4
17-OHCS (μmol/day)	NA	64.2	NA	8.3–33.2
Glucose (mmol/L)	NA	9.07	5.77	3.6–6.1
Calcium (mmol/L)	NA	2.64	1.96	2–2.6

Abbreviations: PRL, prolactin; PTH, parathyroid Hormone; TSH, thyroid stimulating hormone; FSH, follicle-stimulating hormone; LH, luteinizing hormone; TESTO, testosterone; ACTH, adrenocorticotropic hormone; PM: post meridiem; E2: estrogen 2; PROG, progesterone; 17-OHCS, 17-hydroxycorticosteroid; NA, not available.

Patient III-6, the proband's elder son, was diagnosed with a parathyroid tumor and a thoracic tumor at the age of 37. The parathyroid tumor and thoracic tumor were surgically resected and were histologically diagnosed as parathyroid adenoma and thoracic neuroendocrine tumor, respectively (data not shown).

### Novel germline MEN1 splicing donor mutation IVS9 + 1 G > C

**Figure 2:**
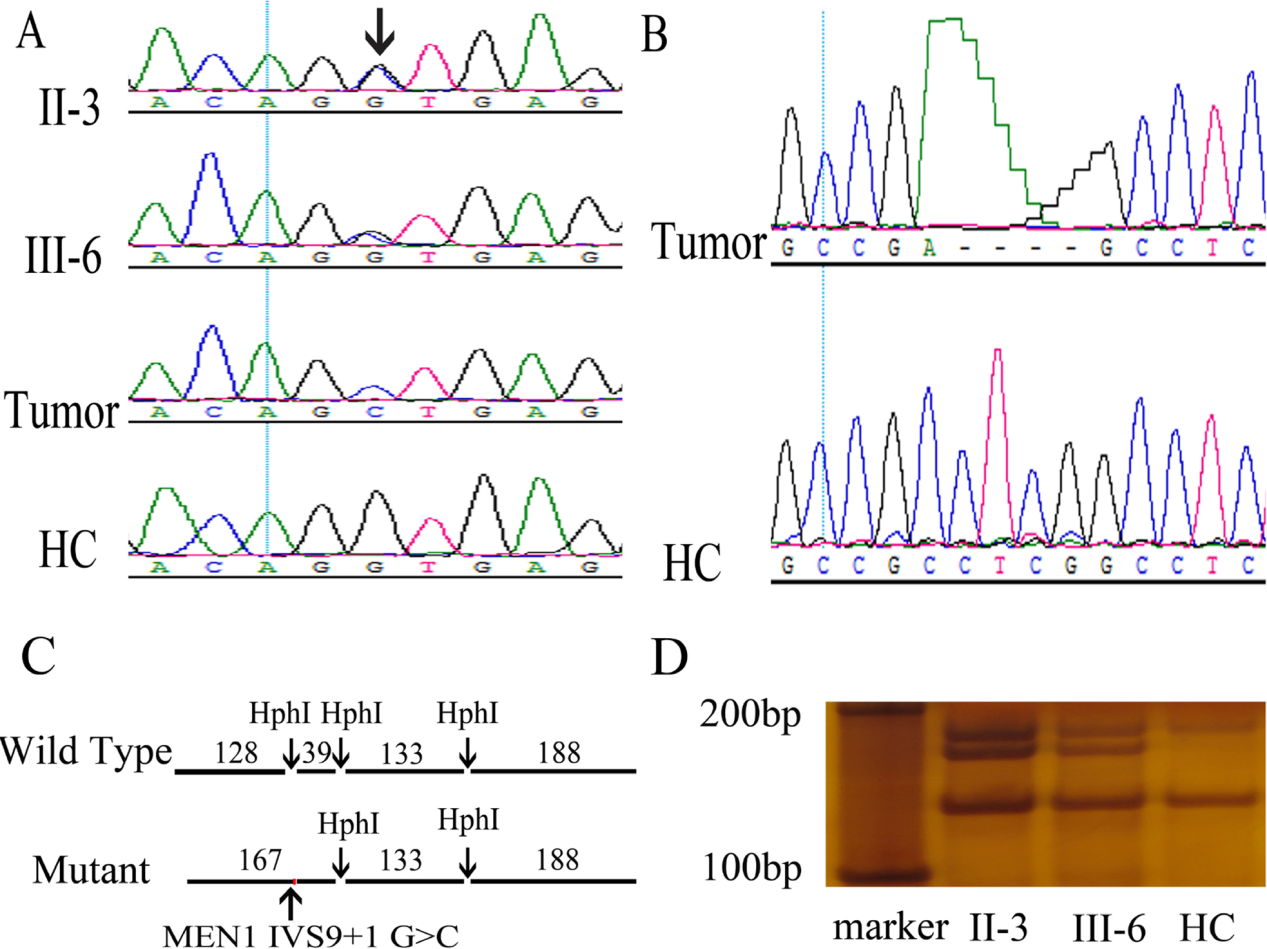
Discovery and validation of two novel *MEN1* mutations, germline IVS9 + 1 G > C and somatic c.1402_1405del GAGG, in the MEN1 syndrome family (**A**) A novel heterozygous germline mutation, *MEN1* IVS9 + 1 G > C, was found in II-3 and III-6 using Sanger sequencing of DNA in peripheral blood leukocytes. Loss of heterozygosity was found in the MEN1 patient tumor using Sanger sequencing of DNA in tumors. Arrow indicates the mutation site. (**B**) A novel somatic mutation *MEN1* c.1402_1405del GAGG was found in tumors of the MEN1 syndrome patients by Sanger sequencing. (**C**, **D**) Restriction fragment length polymorphism confirmed the *MEN1* IVS9 + 1 G > C heterozygous germline mutation in MEN1 patients. *MEN1* gene fragments including IVS9 + 1 were amplified by PCR with primers, E9-3F and E10-3R. The wild-type PCR product had three HphI sites, and HphI digestion generated four fragments with lengths of 128, 39, 133, and 188 bp. However, the mutation IVS9 + 1 G > C was located in an HphI site. The mutant PCR product had only two HphI sites and was cut to three fragments with lengths of 167, 133, and 188 bp. In RFLP analysis, II-3 and III-6 had the additional 167 bp fragments compared with healthy controls (HCs).

Whole exome sequencing (WES) of peripheral blood lymphocyte DNA from both patients revealed 7 single nucleotide variants in the *MEN1* gene. The novel variant NM_130799.2: c.1350 + 1 G > C, or IVS9 + 1 G > C, was not found in the HGMD, dbSNP, 1000G_ALL, and ClinVar databases. The other 6 variants were known benign SNPs in 1000G_ALL. Sanger sequencing confirmed the presence of heterozygous IVS9 + 1 G > C in II-3 and III-6 (Figure 2A). IVS9 + 1 G > C was inherited from father to son and co-segregated with MEN1 syndrome. Furthermore, IVS9 + 1 G > C was not found in other members of this family or 50 HCs. Restriction fragment length polymorphism (RFLP) analysis with HphI verified that II-3 and III-6 had the germline heterozygous IVS9 + 1 G > C mutation (Figure 2C, 2D).

### Analysis of *MEN1* IVS9 + 1 G > C in silico

Bioinformatics analysis was used to evaluate *MEN1* IVS9 + 1 G > C in silico (Table 2). The Fathmm (http://fathmm.biocompute.org.uk/) score of IVS9 + 1 G > C was 0.98943, which indicated IVS9 + 1 G > C mutation was deleterious. In addition, *MEN1* IVS9 + 1 G > C was predicted to be “disease-causing” by MutationTaster (http://www.mutationtaster.org/). The PhyloP (http://compgen.bscb.cornell.edu/phast/) value was 4.559 and the phastCons (http://compgen.bscb.cornell.edu/phast/) value of *MEN1* IVS9 + 1 G > C was 1 in MutationTaster, which indicated that the mutation site was conserved. The in silico analysis of *MEN1* IVS9 + 1 G > C indicated that it was a pathogenic point variant.

**Table 2:** Analysis of *MEN1* mutations in silico

Items	MEN1 IVS9 + 1 G > C, or NM_130799.2: c.1350 + 1 G > C	MEN1 c.1402_1405 del GAGG	MEN1 c.286 C > T
Genome alteration	chr11:64572505 C > G	chr11:64572249_64572252del CCTC	chr11:64577296 G > A
Transcript	Transcript variant SDM ^a^	NM_000244.3: c.1402_1405del GAGG	NM_000244.3:c.286 C > T/ p.Gln96^*^
Mutation type	Germline	Somatic in tumor	Somatic in tumor
Function	Splice donor variant	Frameshift variant	Stop gained variant
Impact	High	High	High
1000G_ALL ^b^	None ^c^	None	None
HGMD ^d^	None	None	None
dbSNP ^e^	None	None	None
ClinVar ^f^	None	None	None
Fathmm ^g^	0.98943	None	0.96778
MutationTaster ^h^	Disease-causing	Disease-causing	Disease-causing
PhyloP ^i^	4.559	4.794	3.424
phastCons ^j^	1	0.998	1

Notes: a: Transcript variant SDM, the abnormal *MEN1* transcript variant caused by IVS9 + 1 G > C splicing donor mutation; b: 1000G_ALL, 1000 Genomes Project; c: None, the mutation was not found in the database or not available in the algorithm; d: HGMD, Human Gene Mutation Database; e: dbSNP, Single Nucleotide Polymorphism database; f: ClinVar, ClinVar database in NCBI; g: FATHMM is an in silico predictive algorithm of the functional consequences. Predictions are given as *p*-values in the range [0, 1]; values above 0.5 are predicted to be deleterious, while those below 0.5 are predicted to be neutral or benign. h: MutationTaster is an in silico predictive algorithm that predicts an alteration as one of four possible types: disease-causing, disease-causing automatic, polymorphism, or polymorphism automatic; i, j: In MutationTaster, PhastCons (range [0, 1]) reflects the probability that each nucleotide belongs to a conserved element, and PhyloP (range [-14, + 6]) measures conservation at individual columns and ignores the effects of their neighbors. The higher the values of PhastCons and PhyloP, the more probable the nucleotide is conserved.

### Functional analysis of *MEN1* IVS9 + 1 G > C

The significance of nucleotide alterations in *MEN1* IVS9 + 1 G > C was determined by RT-PCR. The total RNA was extracted from proband tumor tissues and peripheral blood lymphocytes of III-6 and HC to identify the impact of splicing donor mutation on *MEN1* transcription. Agarose gel electrophoresis of the proband and III-6 RT-PCR products showed an additional larger band migrating slower than the normal band Figure 4A.

**Figure 3:**
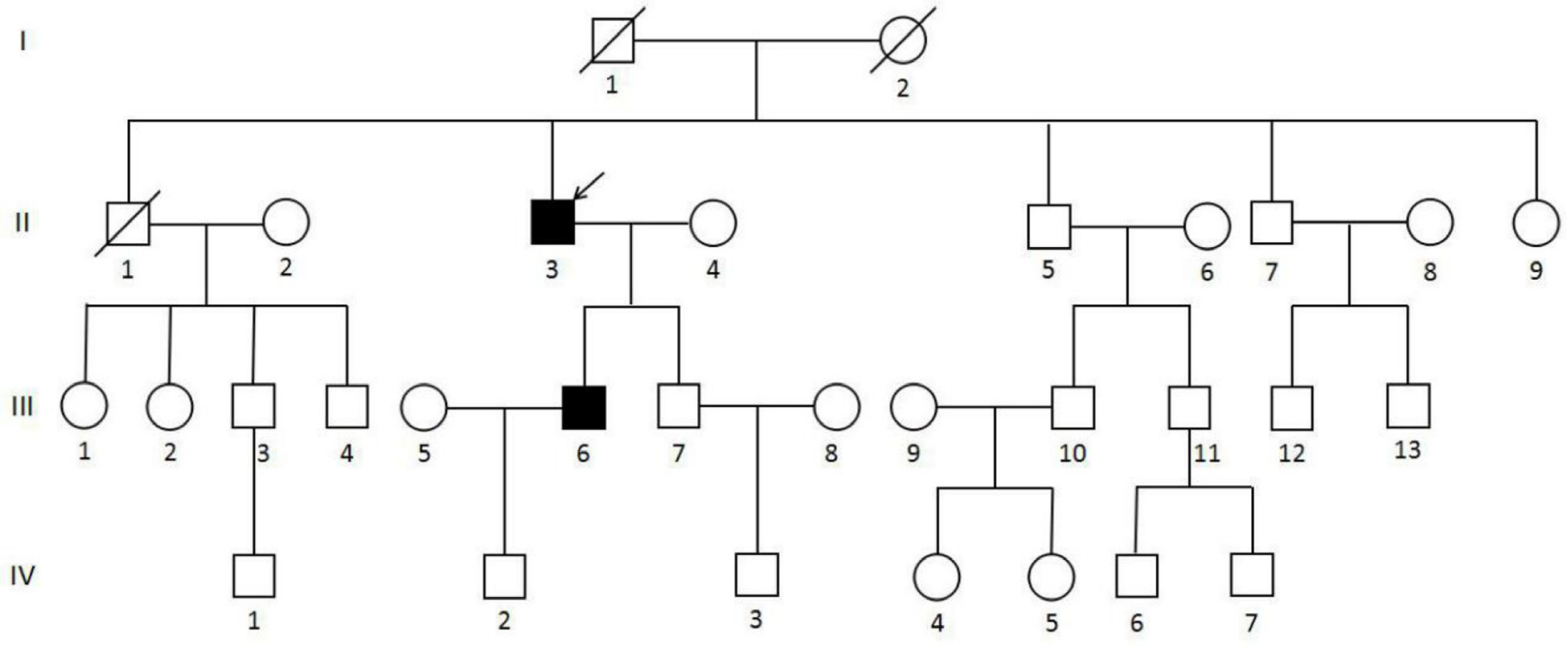
Pedigree of the MEN1 syndrome Han Chinese family with the MEN1 IVS9 + 1 G > C splicing donor mutation Circles indicate women, and squares indicate men. Clinical status is denoted as follows: open symbols, unaffected; solid symbols, affected; slashed, deceased; and arrow, proband.

**Figure 4:**
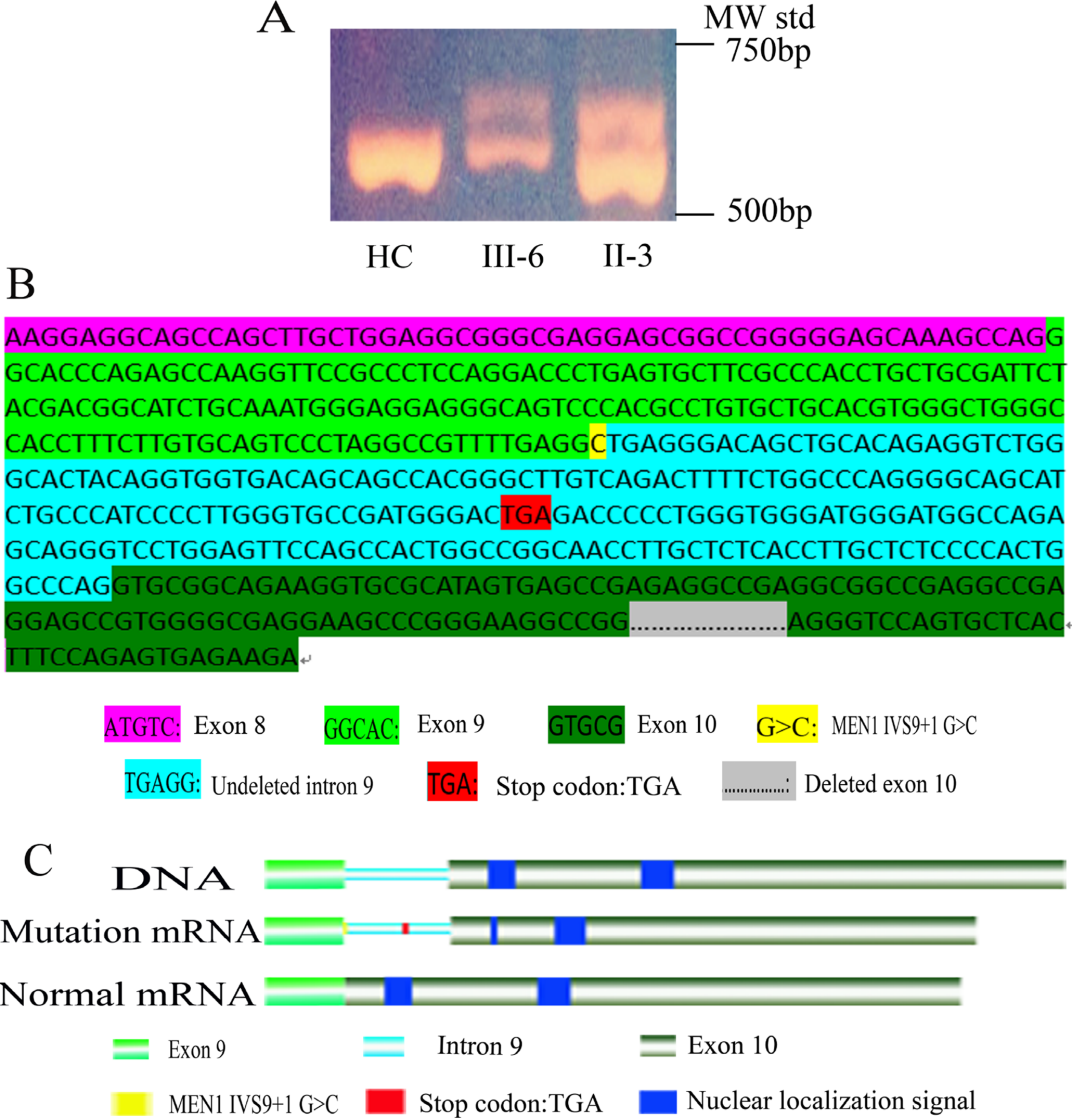
Functional analysis of *MEN1* IVS9 + 1G > C mutation (**A**) In *MEN1* mRNA RT-PCR with primers E8-1F and E10-1R’, II-3 and III-6 had two bands, one normal band and a larger abnormal band, but HC had only one normal band. (**B**) Sanger sequencing of the *MEN1* abnormal mRNA, transcript variant SDM, using RT-PCR and cloning. In the transcript variant SDM, the intron 9 (217 bp) was not deleted, but 202 bp of exon 10 was sheared. In the ORF of transcript variant SDM, the stop codon TGA of intron 9 resulted in the untranslated exon 10 sequence. (**C**) Nuclear localization signals were not translated in transcript variant SDM. IVS9 + 1 G > C resulted in the loss of two nuclear localization signals in menin, presumably disrupting nuclear translocation and functionality.

*MEN1* RT-PCR products were cloned into pGEM-T for Sanger sequencing to obtain the precise sequence. The larger band contained the *MEN1* intron 9 sequence (217 bp), but 202 bp of the *MEN1* exon 10 sequence were sheared (Figure 4B). As a result, the additional band was 15 bp larger than the normal band. The splicing mode of *MEN1* was changed due to the IVS9 + 1 G > C mutation. A transcription variant splicing donor mutation (SDM) was identified and abnormal mRNA was produced in MEN1 patients. We identified a new stop codon TGA in the undeleted *MEN1* intron 9 sequence of the transcription variant SDM open reading frame (ORF), which prevented translation of the *MEN1* exon 10 sequence (Figure 4B). This mutation resulted in the loss of the two NLSs in exon 10 (Figure 4C), which suggested that the mutant menin protein product cannot enter the nucleus for proper functioning [8]. We concluded that this was the mechanistic cause of this MEN1 family.

### Two hit of *MEN1* in parathyroid carcinoma

DNA of the peripheral blood lymphocytes and tumor specimens were sequenced by WES, and the results were compared to identify somatic mutations in MEN1 tumors. A novel *MEN1* mutation, C.1402_1405del GAGG, was found in the parathyroid carcinoma tissue of II-3 and verified by RT-PCR and clone sequencing of *MEN1* mRNA in tumor tissue (Figure 2B). The in silico analysis of this mutation indicated it was a disease-causing frameshift variant (Table 2). In addition, Sanger sequencing revealed the LOH of *MEN1* IVS9 + 1 G > C in the parathyroid carcinoma tissue of II-3 (Figure 2A). The novel MEN1 nonsense mutation, MEN1 c.286 C > T, was found in the formalin fixed paraffin-embedded (FFPE) tissue of thoracic neuroendocrine tumor resected from III-6 by WES. Two additional FFPE DNA samples were poor quality and unsuitable for WES. The in silico analysis of MEN1 c.286 C > T indicated it was a disease-causing nonsense variant (Table 2). The two-hit pathogenic mechanism of MEN1 was revealed by two different somatic mutations in the parathyroid carcinoma tissue of II-3 and a novel somatic mutation in the thoracic neuroendocrine tumor tissue of III-6.

## DISCUSSION

A total of 29 MEN1 mutations have been found in Chinese populations. Long-term follow-up analysis is rarely reported [9–14]. Here, we identified a novel germline-splicing donor mutation and two novel somatic mutations using WES in a Han Chinese MEN1 family with a 6-year follow-up period. A two-hit mechanism was determined to cause MEN1. Our study elucidated the clinical and genetic features of MEN1 in Han Chinese.

The proband was diagnosed with functional pituitary adenoma with high serum prolactin in 2010, but the patient did not elect for surgical treatment and was treated with bromocriptine instead. During the 6-year follow-up he underwent two surgical procedures. The pituitary adenoma was well-controlled and decreased in size. Therefore, MEN1 tumors should be considered curable if the patient is adequately treated by multidisciplinary cooperation. This conclusion was consistent with the results of Ning [15].

Positional cloning of the *MEN1* gene in 1997 enabled the accurate genetic diagnosis of MEN1 and early or presymptomatic diagnosis of at-risk patient relatives, which led to long-term outcome improvement [4, 16]. We conducted genetic screening among a Han Chinese MEN1 family, and a novel splicing donor germline mutation (IVS9 + 1 G > C) was discovered in the proband and one of his sons. This novel mutation caused the retention of intron 9 and deletion of exon 10 during splicing that resulted in the early stop of *MEN1* translation with subsequent truncation of the normal menin protein. *MEN1* intron 9 had additional splicing mutations, *MEN1* IVS9 + 1 G > A [17] and IVS9-1 G > T [19], that caused the retention of intron 9 and resulted in an ORF frameshift of *MEN1* similar to IVS9 + 1 G > C. However, the clinical manifestation of IVS9 + 1 G > C varied from that of the other two mutations. IVS9 + 1 G > A patients manifested familial isolated primary hyperparathyroidism and tested negative for multiple endocrine neoplasia. IVS9-1 G > T patients manifested familial isolated primary hyperparathyroidism. IVS9 + 1 G > C patients presented pituitary adenoma, pancreatic tumor, adrenal cortical tumor, abdominal lipoma, thoracic neuroendocrine tumor, and parathyroid adenoma. In addition, *MEN1* IVS9 + 1 G > C and G > A had the same mutation site with dissimilar clinical features. In contrast to the studies of Ning and Raghavan, this result indicated that a different missense mutation at the same site could lead to a different phenotype [15, 18].

MEN1 occurrence could be explained by Knudson's two-hit theory. In the dominantly inherited form, one mutation is inherited via the germinal cells and the second via somatic cells [20]. According to Thakker et al., the sequential inactivation of the *MEN1* gene is an essential step in the development of MEN1 tumors [21]. The novel somatic mutation, *MEN1* c.1402_1405del GAGG, and LOH was identified in the parathyroid carcinoma tissue of II-3, and a novel somatic mutation, MEN1 c.286 C > T, was found in the thoracic neuroendocrine tumor of III-6. This result supported the two-hit mechanism of MEN1 development.

We identified the clinical features of a Han Chinese MEN1 family within a 6-year follow-up period and the novel splicing donor and somatic mutations by WES. Our study illuminated the genetic mechanism, clinical features, and management of Chinese MEN1 patients.

## MATERIALS AND METHODS

### Subjects and samples

A Han Chinese MEN1 family (Figure 3), including 4 generations and 31 members, received follow-up study since August 2010 to September 2016 in Xiangya Hospital, Hunan, Changsha, China. The proband visited Xiangya Hospital in 2010 at the age of 65 and was diagnosed with MEN1 in following medical inspections. The proband's son also showed clinical symptoms of MEN1 at the age of 37. Other members of this family were not affected by MEN1. To confirm the presence of the mutation in the family, 50 healthy controls (HCs) from the medical examination center of Xiangya Hospital were also enrolled in the present study. Peripheral blood or saliva was collected from all members of the MEN1 family and HCs. Approximately 2 mL of peripheral blood was collected in tubes containing anticoagulant EDTA-K2. Saliva was stored in tubes containing 70% alcohol. Surgical resection of a parathyroidal tumor of II-3 was collected in the frozen tube and stored at -80°C. FFPE tumor tissue of pancreatic of II-3 and the thoracic neuroendocrine gland and parathyroid of III-6 were collected. The experiments were conducted in accordance with the Declaration of Helsinki (2013). Informed consent was obtained from the families and 50 healthy participants in this study according to the Declaration of Helsinki (2013) and the protocol was approved by the Research Committee of Xiangya hospital.

### WES and bioinformatics analysis

WES was performed using DNA isolated from leukocytes of II-3 and III-6 and the parathyroid carcinoma specimen of II-3 with the Dzup kit (Dzup, Sangon Biotech Co. Ltd, Shanghai, China), and the other three paraffin-embedded tumor tissues with the QIAGEN kit (QIAGEN, Sample & Assay Technologies, Germany). Sequencing libraries were constructed using a modified Illumina HiSeq X-Ten DNA sample preparation kit (BGI, Shenzhen, China). Qualified genomic DNA sample was randomly fragmented by the Covaris Acoustic System, and adapters were ligated to both ends of the resulting fragments. Extracted DNA was amplified by ligation-mediated PCR (LM-PCR), purified, and hybridized to the Nimblegen SeqCap EZ Library v3.0 (Roche/NimbleGen, Madison, WI) for enrichment. Both non-captured and captured LM-PCR products were subjected to quantitative PCR to estimate the magnitude of enrichment. Each captured library was loaded on the Hiseq2500 platform (Illumina, San Diego, CA). We performed high-throughput sequencing for each captured library to ensure that each sample met the desired average sequencing depth (903). Raw image files were processed by Illumina base-calling Software 1.7 for base-calling with default parameters, and the sequences were generated as 90 bp pair-end reads. Single nucleotide polymorphisms (SNPs) and insertion/deletions were called using the Genome Analysis Toolkit (GATK v1.5.11). Novel mutations were determined by searching the Human Gene Mutation Database (HGMD), dbSNP, 1000 Genomes Project data (1000G_ALL) and ClinVar database. The functions of *MEN1* mutations were analyzed using predictive algorithms in silico, such as MutationTaster (http://www.mutationtaster.org) and Fathmm (http://fathmm.biocompute.org.uk) [22, 23].

### PCR sequencing of *MEN1*

*MEN1* fragments containing the IVS9 + 1 site were amplified by PCR with primers E9-1F (5′-ACCTGCTGCGATTCTACGAC-3′) and E10-1R (5′-ACTATGCGCACCTTCTGCC-3′). PCR was performed in a total volume of 20 μL, including 0.3 μL DNA (15 ng), 0.3 μL forward primer (20 μmol/L), 0.3 μL reverse primer (20 μmol/L), 10 μL PrimeSTAR (Takara Biotechnology Co, Ltd. Japan), and 9.1 μL deionized water. The PCR program was as follows: 98°C 5 min; 98°C 10 s, 58°C 5 s, and 72°C 1 min for 40 cycles, followed by 72°C for 5 min. The PCR products were purified and used for Sanger sequencing (Biosune, Shanghai, China).

### Restriction fragment length polymorphism

*MEN1* fragments containing the IVS9 + 1 site were amplified by PCR with primers E9-3F (5′-ACCTGCTGCGATTCTACGAC-3′) and E10-3R (5′-TTGTCCAGTGCTGGCTTCTT-3′). The PCR products were separated by agarose gel electrophoresis, and the *MEN1* fragments were purified using a SanPrep DNA gel extraction kit (Sangon Biotech Co. Ltd, Shanghai, China). *MEN1* fragments were digested with HphI restriction enzyme (Thermo Scientific, USA), which recognizes 5′ –GGTGA (N) 8↓- 3′. The reaction consisted of 18 μL nuclease-free water, 2 μL 10× Buffer B, 10 μL DNA (0.5 μg/ μL), 1 μL HphI (10 U/μL). The reaction was incubated at 37°C for 1.5 h and then at 65°C for 20 min. The digested products were separated by 6% non-denaturing polyacrylamide gel electrophoresis at 250 V for 3 h and visualized by silver nitrate staining.

### Total RNA extraction, RT-PCR, and cloning

Total RNA was extracted from frozen tumor tissues and fresh peripheral blood cells using TriZol Reagent (Ambion, USA). Frozen tumor tissues were pulverized with liquid nitrogen before TriZol treatment. Fresh peripheral blood cells were separated by lymphocyte separation medium (TBD, Tianjin, China) to obtain lymphocytes. Reverse transcription (RT) was performed with total RNA using the Revert Aid First-Strand cDNA Synthesis Kit (Thermo Scientific, USA). *MEN1* cDNA fragments were then amplified by PCR with primers E8-1F (5′-ATGTCATCCCCAACCTGCTG-3′) and E10-1R’ (5′-CAGCTCCTTCATGCCCTTCA-3′), which were designed based on the sequences spanning from exon 8 to exon 10. Finally, RT-PCR fragments were cloned into pGEM-T (Promega, Beijing, China) for Sanger sequencing (Biosune, Shanghai, China).
